# Mortality trajectory analysis reveals the drivers of sex-specific epidemiology in natural wildlife–disease interactions

**DOI:** 10.1098/rspb.2014.0526

**Published:** 2014-09-07

**Authors:** Jennifer L. McDonald, Graham C. Smith, Robbie A. McDonald, Richard J. Delahay, Dave Hodgson

**Affiliations:** 1 Centre for Ecology and Conservation, University of Exeter, Penryn, Cornwall TR10 9EZ, UK; 2 Environment and Sustainability Institute, College of Life and Environmental Sciences, University of Exeter, Penryn, Cornwall TR10 9EZ, UK; 3 National Wildlife Management Centre, Animal Health and Veterinary Laboratories Agency, Sand Hutton, York YO41 1LZ, UK

**Keywords:** sex differences, disease, Bayesian, survival analysis, badgers, tuberculosis

## Abstract

In animal populations, males are commonly more susceptible to disease-induced mortality than females. However, three competing mechanisms can cause this sex bias: weak males may simultaneously be more prone to exposure to infection and mortality; being ‘male’ may be an imperfect proxy for the underlying driver of disease-induced mortality; or males may experience increased severity of disease-induced effects compared with females. Here, we infer the drivers of sex-specific epidemiology by decomposing fixed mortality rates into mortality trajectories and comparing their parameters. We applied Bayesian survival trajectory analysis to a 22-year longitudinal study of a population of badgers (*Meles meles*) naturally infected with bovine tuberculosis (bTB). At the point of infection, infected male and female badgers had equal mortality risk, refuting the hypothesis that acquisition of infection occurs in males with coincidentally high mortality. Males and females exhibited similar levels of heterogeneity in mortality risk, refuting the hypothesis that maleness is only a proxy for disease susceptibility. Instead, sex differences were caused by a more rapid increase in male mortality rates following infection. Males are indeed more susceptible to bTB, probably due to immunological differences between the sexes. We recommend this mortality trajectory approach for the study of infection in animal populations.

## 1. Introduction

There is increasing epidemiological evidence of sex-related differences in host–pathogen interactions in animal populations. Males are usually more likely than females to acquire infection and die from disease once infected [1]. However, the mechanisms that drive these sex biases remain poorly understood. By changing our view of mortality parameters, from fixed rates in discrete stage classes to mortality trajectories, we aim to deconstruct the mortality process in infected males and females, uncovering when in the infection process sex differences arise, and helping to identify the mechanisms that generate such variation.

The most obvious driver of sex differences in infectious disease-induced mortality is that disease affects males more than it does females due to weaker, or simply different, physiologies [2]. Genetic differences between the sexes may directly impact upon disease susceptibility, with sex-linked genes known to be determinants of immune functioning [3]. Sex hormones have also been linked to male-biased mortality due to their role in determining immunocompetence [4]. Androgens, in particular testosterone, are known to regulate male reproductive trade-offs [5,6], suppressing disease defences [7,8]. Indirect mechanisms of sex differences include the possibility that infection itself causes sex-biased changes in behaviour (e.g. increased fighting or ranging), exposing males and females to differential risk of mortality as infections progress.

Alternatively, behavioural and ecological differences between the sexes might indirectly make males simultaneously more likely to acquire infection and die from other causes. In many species, including humans [9], males are more likely to engage in risk-taking behaviours, increasing their disease exposure. Risky behaviours such as higher levels of aggression [10] and wider-ranging movements [10,11] may simultaneously elevate male mortality and increase infection risk, giving rise to a correlation between infection risk and increased mortality, but no direct causality.

A third potential driver of sex differences is that infection may have disproportionate effects on substandard males that are already in poor body condition, resulting in observable differences in heterogeneity in response to infection between the sexes. In this case, maleness is simply a proxy for susceptibility to disease: the true driver is poor body condition, but more males than females tend to be in this state.

Classical statistical approaches to demographic or epidemiological analysis of surveys of wild animal populations tend to estimate fixed mortality parameters for pre-defined classes of population members (e.g. male versus female; young versus old). Fixed mortality parameters assume that infected individuals experience an exponential decay in the numbers surviving over the infection period, and thus fail to consider infection as a more complex, time-varying process. Fixed mortality parameters also fail to reveal immunological or behavioural causes. In reality, mortality trajectories will be more complicated than the exponential process, and differences in the parameters of mortality curves among classes of individuals can reveal important epidemiological processes. If males are coincidentally more likely to develop disease and die of other causes, we predict elevated male mortality at the point of infection. If males are more susceptible to disease because the male class harbours the greater share of substandard individuals, we predict less heterogeneity in disease-induced mortality among males than among females. Finally, if males are genuinely more susceptible to disease-induced effects, we predict that the rate of increase in mortality, post-infection, will be greater in males than in females.

One reason for the paucity of time-varying mortality trajectories of infected hosts in wild populations is that individuals cannot be monitored continuously from time of infection to death. However, age-specific mortality functions are commonly used in human and wildlife demographic analyses, and a recently developed method can estimate age-specific mortality trajectories while accommodating uncertainty in dates of birth and death [12]. We employ this Bayesian survival trajectory framework (BaSTA [13]) to describe disease-induced mortality trajectories, accounting for uncertainty in date of infection, and apply this method to obtain mortality patterns for different health states in a population of wild badgers (*Meles meles*) naturally infected with *Mycobacterium bovis*, the causative agent of bovine tuberculosis (bTB).

Sex differences in epidemiological traits have been observed in bTB-infected badgers, with males suffering increased mortality during early disease stages and faster progression into advanced diseased states [14], where they experience double the rate of disease-induced mortality when compared with females [14,15]. We test contrasting hypotheses and describe sex-related differences in the mortality trajectories of badgers. Given the economic importance and high public profile of badgers as a reservoir of bTB [16], it is critical that we better understand the epidemiology of this disease. Teasing apart the behavioural, ecological and physiological drivers that divide the wildlife population into categories of susceptibility to disease may inform improved strategies to reduce rates of transmission to livestock [17].

In summary, we have applied a new methodology for analysing longitudinal demographic data, which provides mortality trajectories rather than discrete rates of mortality during different stages of disease progression. We suggest that this methodology can be used to obtain mortality trajectories that depend on infection duration, rather than age *per se*. We show that bTB infection alters mortality trajectories of badgers. We describe differences in mortality trajectories between uninfected and infected states, and, by focusing attention on variation over time, the role of sex in shaping heterogeneity in disease response. The ability of BaSTA to account for unknown date of infection provides opportunities to explore disease-specific mortality trajectories in this and other wild mammal populations, paving the way for a better understanding of the role of sex in epidemiology.

## 2. Material and methods

### (a) Ecological data

We used capture–recapture data collected from an intensively studied natural population of badgers in Woodchester Park, Gloucestershire, UK for the period 1984–2005. Twenty social groups that were trapped consistently throughout the study period were incorporated in this analysis. Badgers were trapped approximately quarterly. They were anaesthetized and each was given a unique identifying tattoo on its first capture (for detailed methods, see [18]). Blood samples were taken and tested for antibodies to *M. bovis* using an enzyme-linked immunosorbent assay (the Brock ELISA [19]). Samples of faeces, urine, sputum and pus from abscesses and/or bite wounds (where relevant) were taken for bacterial culture of *M. bovis* [20,21]. Badgers were categorized according to these diagnostic test results as either uninfected (U), defined as a test-negative badger, or infected (I), including badgers that test positive to the ELISA test and/or culture. In our categorization, we made two assumptions. First, as bTB is a progressive disease in badger populations, once classified as infected we assumed that a badger did not recover (in accordance with previous studies [18,22]). Second, we assumed accuracy of diagnostic tests. Accurate diagnosis in live badgers is difficult due to limitations in the performance of the ELISA test, which has a specificity of 89–94% [23,24] and culture which, despite high specificity, has low sensitivity [25]. Violation of these assumptions due to error in ascribing infection status would only act to weaken the signal of mortality effects in infected badgers, thus making our results conservative. Individual quarterly capture histories were created for uninfected and infected badgers with sex incorporated as a covariate, totalling 7957 capture occasions across 1460 individual capture histories for 786 females (125 of which were ‘infected’) and 674 males (124 of which were ‘infected’). Survival analysis was then applied to the separate datasets.

### (b) Modelling framework

To account for uncertainty in infection date, we fitted BaSTA [13] to capture data for infected badgers using the software R [26]. BaSTA uses a capture–mark–recapture approach incorporating recapture probabilities less than one, thereby providing a powerful analysis that can account for variable recapture rates. Recapture probability was kept fully time-dependent throughout the analysis, accounting for any temporal recapture bias.

BaSTA models ‘birth’ years (in this case, year of infection) and death years as latent variables, drawing inference on age- or time-since-infection-specific mortality despite missing data. For the uninfected badgers analysed, prior information on the year of birth was obtainable when badgers were first caught as cubs or yearlings, therefore under such circumstances birth dates were incorporated, consisting of 1011 known birth dates. With regard to the infected badgers, we cannot be certain when an individual entered a diseased state, therefore no date was included. Dates of death were recorded when badgers were found dead: time of death was known for 214 uninfected badgers and 48 infected badgers.

Four mortality functions, each able to describe different trends in mortality, were compared [13]:

(i) *Exponential*. The simplest trajectory models consist of a single constant mortality parameter that assumes mortality is independent of the duration of infection, equivalent to the fixed discrete rates we commonly see in wildlife disease analyses.
(ii) *Gompertz*. These models consist of two parameters; an initial mortality and an exponential increase in mortality parameter [13].
(iii) *Weibull*. This model has two parameters, a shape and a scale [13,27]. The versatility of the model means it can show accelerating increase, decelerating increase, and decreasing or constant mortality.
(iv) *Logistic*. This model has three parameters [13]. It is similar to a Gompertz model with an additional deceleration parameter whereby mortality levels off over time. In terms of mortality trajectories of an infected population this levelling off could represent a reduction in mortality at advanced duration of infection (i.e. an improvement in survival), or (more likely) heterogeneity in disease response [28].

To ensure model convergence, initial trials of four Markov chain Monte Carlo (MCMC) iterated samplings (chains) were run for each model, followed by 100 000 iterations on four chains with a burn-in of 20 000 for each analysis. Convergence was assessed both visually ensuring mixing of the chains and formally within the model calculating the potential scale reduction (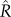 [13]). When 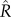 is close to 1 we can be confident that convergence has been reached; the burn-in period was determined when 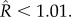 We also tested mortality parameters for prior sensitivity, running the model for both uninfected and infected badgers under four different prior structures. The choice of prior did not influence the identification of mortality parameters or differences among them. The deviance information criterion (DIC) [29] was used to assess model fit. Additionally, BaSTA provides a diagnostic tool based on Kullback–Leibler discrepancies [30] calibrated to reduce asymmetry (KLDC), which provides an assessment of the extent of overlap of posterior distributions of parameter estimates for categorical variables. This is a value between 0.5 and 1: a value of 0.5 indicates identical distributions, and 1 that there is no overlap between them [13]. This allows us to determine the magnitude of the effect of sex on the parameters of mortality trajectories.

### (c) Predictions

**Figure 1.**
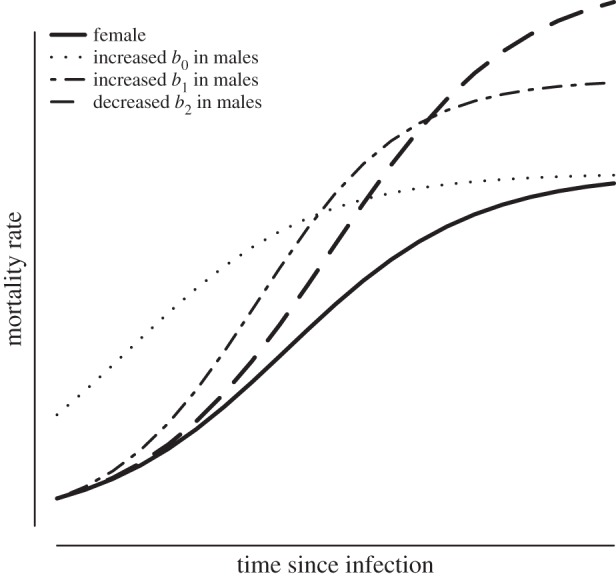
Hypothetical, sex-specific, logistic mortality trajectories driven by different mechanisms. Hypothesis 1: the sexes are differentiated by rates of mortality at the point of infection (*b*_0_). Hypothesis 2: sexes are differentiated by the rate of increase in mortality post-infection (*b*_2_). Hypothesis 3: sexes are differentiated by their degree of deceleration post-infection, an artefact of heterogeneity in disease response.

Using a logistic model to represent mortality trajectories following infection (figure 1), we formulated hypotheses regarding the cause of the established sexual dimorphism in infection response among badgers. The logistic model relates mortality rates (*μ*) to time since infection (*x*),2.1
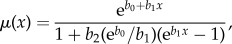
in which *b*_0_ represents mortality at the point of infection, *b*_1_ describes the rate of mortality increase post-infection and *b*_2_ highlights deceleration in mortality rates.

Hypothesis 1. If sex differences in mortality are caused by a coincidental predisposition to die and to also become infected, we would expect to find differences in mortality at the point of infection (*b*_0_; figure 1).
Hypothesis 2. If sex differences are caused directly by disease, we would expect similar intercept values (*b*_0_) but sex-related differences in the subsequent rate of increase in mortality post-infection (*b*_1_; figure 1).
Hypothesis 3. If maleness is a proxy for susceptibility to disease with the male sex harbouring a greater proportion of substandard individuals, we would expect reduced heterogeneity in response, indicated by a reduction in the deceleration parameter (*b*_2_; figure 1). It should also be noted that if the male sex tends to harbour substandard individuals, we might also observe higher male mortality at the point of infection (hypothesis not graphed).

## 3. Results

**Figure 2.**
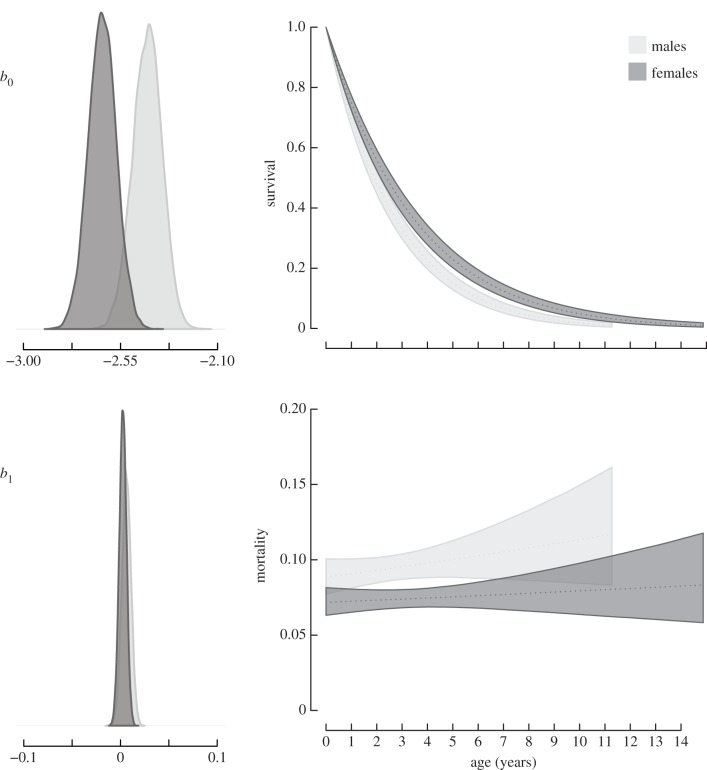
Age-dependent survival and mortality trajectories of uninfected male and female badgers. Initial mortality values (*b*_0_) at point of birth were higher for males than females, but the rate of mortality increase (*b*_1_) was similar between the sexes. Uninfected mortality trajectories were best described by Gompertz functions.

**Figure 3.**
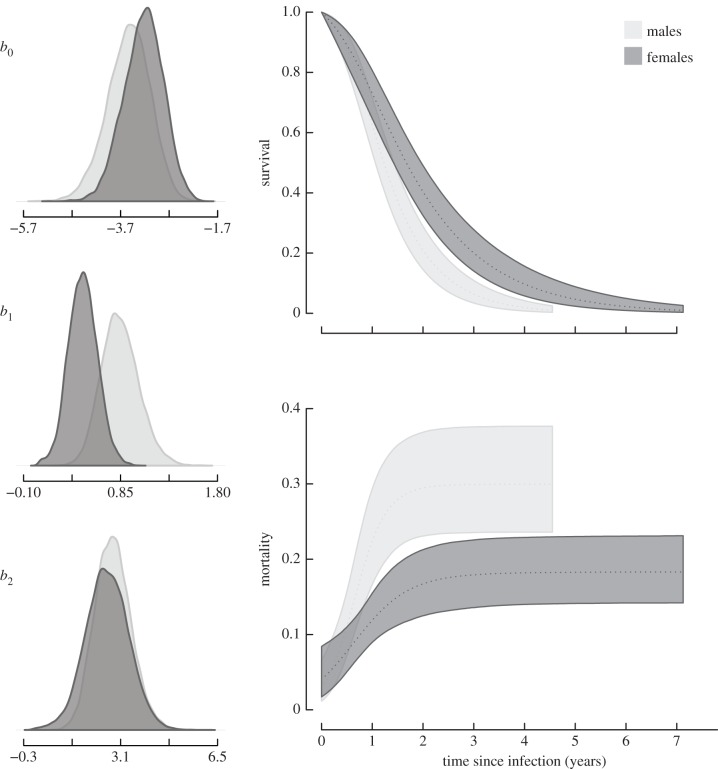
Logistic survival and mortality trajectories of badgers following infection. At the point of infection, there is no discernible difference between sexes (*b*_0_); however, males have elevated rates of increase in mortality following infection (*b*_1_), and males and females display similar levels of heterogeneity (*b*_2_) in disease responses.

The Gompertz model was most supported for uninfected badgers (table 1 and figure 2), consisting of just two parameters: initial mortality at the point of birth (*b*_0_) and the rate of mortality increase (*b*_1_). Once infected, the logistic mortality function (equation (2.1)) was most supported, consisting of an additional deceleration parameter (*b*_2_; table 1 and figure 3). As the use of DIC values has been considered controversial [12,31], further support for a logistic trajectory in infected badgers is provided in table 2, wherein *b*_2_ is identifiably different from zero, upholding the rejection of the simple Gompertz model. These results were robust under four different prior structures (Gompertz priors *b*_0_, *b*_1_: (3, 0.01), (−3, 1), (−2, 1), (−2, 0.01); Logistic priors *b*_0_, *b*_1_, *b*_2_: (−3, 0.01, 0), (−3, 1, 0.01), (−3, 1, 1), (−2, 1, 0.01)).

**Table 1.** Candidate mortality functions for mortality trajectories of male and female badgers in two health states (infected and uninfected), and their corresponding differences in deviance information criterion (ΔDIC). Substantial support for the ‘best’ model alone is indicated when rival models all have ΔDIC > 3 [29].

mortality function	uninfected	infected
exponential	9.1	26.7
Gompertz	0^a^	49.4
logistic	21.5	0^a^
Weibull	29.4	6.7

^a^The most supported model.

**Table 2.** Posterior means and 95% credible intervals of mortality trajectory parameters for uninfected and infected badgers, including intercept (*b*_0_), mortality increase rate (*b*_1_) and for infected badgers a deceleration parameter (*b*_2_).

		uninfected			infected		
		mean	lower 95%	upper 95%	mean	lower 95%	upper 95%
*b*_0_	male	−2.426	−2.56	−2.297	−3.538	−4.464	−2.721
	female	−2.635	−2.762	−2.507	−3.231	−4.064	−2.477
*b*_1_	male	0.006	−0.003	0.015	0.847	0.513	1.238
	female	0.002	−0.005	0.01	0.481	0.202	0.768
*b*_2_	male	—	—	—	2.833	1.682	4.147
	female	—	—	—	2.626	1.122	4.104

Inferred life expectancies decreased once badgers became infected. Life expectancies were consistently shorter in males than in females. To the nearest month, male average life expectancies were estimated to be 32 months for uninfected badgers and 22 months for infected badgers. Female average life expectancies were found to be 40 months for uninfected badgers and 35 months for infected badgers. When they are uninfected, this equates to males having on average a 20% lower life expectancy compared with females, with the acquisition of infection increasing this difference to 37%.

Sex-related differences among uninfected badgers were due to higher initial mortality parameter values in males, suggesting that they are predisposed at birth to have a higher initial mortality than females (KLDC: 1; figure 2 and table 2). Their subsequent lifetime rate of mortality increase was similar to that of females, with a high degree of overlap between posteriors (KLDC: 0.68; figure 2 and table 2).

Infection alters mortality patterns in badgers, with trajectories of infected animals supported by a logistic framework, consisting of an additional deceleration parameter (*b*_2_). At the point of infection, there is no identifiable difference in mortality between the sexes, with significant posterior overlap (KLDC: 0.62; figure 3 and table 2), suggesting that infected males do not represent a biased subset of more susceptible individuals in the population. Following infection, the mortality rate in males increases substantially faster than in females (KLDC: 0.96; figure 3 and table 2). The degree of deceleration or heterogeneity was similar in males and females once infected (KLDC: 0.54; figure 3 and table 2), indicating a similar spectrum of responses to disease in both sexes. The absence of the deceleration parameter (*b*_2_) in the trajectories of uninfected individuals suggests that infection promotes an increase in heterogeneity in mortality among badgers (i.e. a wider spectrum of mortality responses). These results support our hypothesis 2, that differences between male and female infected badgers are due to a substantial difference in the post-infection rate of increase of mortality (*b*_1_).

## 4. Discussion

While increased mortality due to bTB infection is already known to occur in male badgers [14,15], we have now located where in the infection process these sex-related differences arise, and can begin to infer the mechanisms that might generate this variation. Mortality rates at the point of infection are very similar between the sexes, suggesting that elevated mortality in infected males is not due to the coincidental risks of natural mortality and infection. We also found no evidence that infected male and female badgers differ in the degree of heterogeneity among individuals in their responses to infection. Instead, the distinction we see between the sexes is that males experience a faster rate of increase in mortality with increasing time since infection. This suggests a difference in immunological or other physiological response to bTB infection between males and females, and/or that infection itself might cause male badgers to behave in ways that increase their risk of death.

Uninfected badgers do demonstrate sex-based differences in mortality and senescence-related mortality. However, the increase in mortality with age in the best-supported Gompertz framework is not substantial compared with other mammals [12] (i.e. evidence of senescence is weak among uninfected wild badgers). Uninfected males are predisposed to higher mortality from the time of birth, resulting in a 20% shorter life expectancy. Possible explanations include increased competitive encounters and bite wounding among males [10], a phenomenon also found in other mammals [32], and/or that males may suffer more than females from other diseases. Uninfected badgers have very different mortality trajectories to infected badgers, characterized by substantially lower mortality rates. Infection with bTB clearly alters prognoses of life expectancy and exaggerates sex differences in survival rates.

Mortality trajectories of infected badgers were best described by logistic curves, whereby after an increase in mortality the trajectory decelerates and reaches a plateau. Although this pattern implies that susceptibility to disease-induced mortality declines with duration of infection in all infected badgers, a rival and more biologically plausible explanation is heterogeneity in individual response to infection [28], whereby the most susceptible individuals die early in the infection process, leaving the more resilient to die later. Male and female badgers show similar levels of heterogeneity in mortality rates. This indicates a comparable spectrum of immune responses, and suggests that a broad array of individuals of both sexes become infected rather than just a biased sample of males that are already predisposed to high mortality rates.

We suggest that sex-related variation in immunocompetence is likely to be the main mechanism for observed differences between the epidemiology of bTB in males and females. Mortality patterns highlight raised mortality in males following infection, but otherwise comparable trajectories, suggesting weaker immunological defences. This is consistent with results of prior studies that showed males suffering rapid disease progression [14] and substantial weight loss [33] following infection. Immunological defences are costly and can trade off with other physiological processes [14,34,35], perhaps resulting in differential investment between the sexes. Although chromosomal differences and other physiological processes cannot be discounted, sex hormones are suggested to be strong determinants of immune response [36], responsible for sex-specific differences to mycobacterial infection across study species [3]. Male immune suppression is commonly found in other mammals [37,38], whereby the cost of allocating resources to reproductive activity (e.g. male ornamentation [39], singing [40], territorial behaviour [41] and aggressive encounters [42]) suppresses immune defences. Such a trade-off, also known as the immunocompetence handicap [7], may explain why male badgers are more likely to become infected and die from bTB [14,15]. The investment in reproductive effort in male badgers is not expressed as extravagant ornamentation, as in some species [7], but more likely by competitive and/or aggressive behaviour [10], maintaining territories, ranging behaviour [22] and the associated investment in a larger body size compared to females [43]. We speculate that investment in growth and reproduction in male badgers may contribute more to fitness than investment in immunological defence against diseases such as bTB.

An intriguing additional (and not exclusive) explanation for higher rates of increase in mortality risk in male badgers is the possibility that infection itself causes changes in behaviour that increase the likelihood of death. Pathogens can manipulate host behaviour [44,45], increasing risk-taking behaviours such as aggression in order to increase physical contact and transmission opportunities between individuals. The possibility that infection drives behavioural changes in male badgers cannot be discounted, with increased aggression one suggested mechanism explaining why infectious male badgers are more likely to be bitten [46], reducing their survival. However, determining the causality is problematic as higher prevalence of bite wounds may also be due to disease-driven reductions in body condition impacting the social status [10] and competitive ability [46] of infected males.

This confirmation that male badgers suffer elevated rates of increase in disease-induced mortality might help inform management strategies designed to reduce bTB prevalence in the wildlife reservoir, or to reduce rates of transmission between badgers and cattle. However, the dynamics of bTB in wildlife and livestock are sufficiently complex [47] that management decisions should be based on broader ecological and epidemiological models rather than individual epidemiological parameters.

We uncover the counterintuitive result that males have similar mortality to females upon becoming infected, despite having higher natural mortality. This may be due to earlier onset of infection in males. That is, males have higher probability of infection [14] and therefore tend to become infected younger, when natural rates of mortality are lower. This would yield overlap in mortality at point of infection between younger males and older females. An alternative explanation may be that females with high mortality, due to other mortality pressures, have increased infection risk. Generally, females have reduced susceptibility to infection, therefore those under additional pressures or co-morbidities (e.g. due to nutritional stress and/or reproduction-mediated drops in immunity [48]) may be at higher risk of infection. Given that multiple mechanisms may drive similar patterns, an individual-level disease analysis may be useful to observe drivers of bTB in female badgers. Unfortunately, there is no current means to incorporate time-varying individual covariates within the BaSTA framework, but such a development would allow variation in body condition and reproductive status to change over time, addressing these questions.

Understanding how the risk of mortality changes as infection progresses provides a key to explaining and predicting the population dynamics of infected hosts, and ultimately informs the development of better intervention strategies for disease control. We demonstrate the utility of a Bayesian modelling framework, developed specifically for age-related survival analysis, but translated here for the analysis of disease-induced mortality trajectories in wildlife populations. Trajectories, as opposed to discrete rates of mortality, can highlight heterogeneity in disease response and stages of maximum vulnerability, and allow comparison of mortality trends between cohorts and classes of infected hosts. Trajectory analysis has revealed key sex-related differences in bTB epidemiology in badgers, and we recommend its application to surveys of disease-induced mortality in other populations and species.

## Data accessibility

For those interested in collaborating in the use of these data, email dez.delahay@ahvla.gsi.gov.uk.
